# Identification and In Vitro Functional Characterization of the *CCR* Gene Family Reveals Their Regulatory Roles in Lignin Biosynthesis of *Pinus yunnanensis*

**DOI:** 10.3390/plants15152253

**Published:** 2026-07-23

**Authors:** Jun Liu, Heze Wang, Jianhong Chang, Aiqin Yao, Junrong Tang

**Affiliations:** 1 Key Laboratory for Forest Resources Conservation and Use in the Southwest Mountains of China, Ministry of Education, Southwest Forestry University, Kunming 650224, China; liujun0102123@163.com (J.L.);; 2 Yunnan Provincial Key Laboratory for Conservation and Utilization of In-Forest Resource, Southwest Forestry University, Kunming 650224, China

**Keywords:** *Pinus yunnanensis*, cinnamoyl coenzyme A reductase, lignin biosynthesis, enzymatic assay in vitro, molecular docking

## Abstract

Cinnamoyl coenzyme A reductase (CCR) is the first rate-limiting enzyme in the monolignol-specific pathway and plays a pivotal role in lignin biosynthesis. However, *CCR* genes in *Pinus yunnanensis* remain uncharacterized, and their undefined substrate specificity further impedes mechanistic insights into lignin regulation while restricting strategies for wood property optimization. Using conserved domain and homology analysis, with a focus on the characteristic NAD(P)-binding motif (KNWYCYGK), we identified 12 CCR family members from the transcriptome data of *P. yunnanensis*. Phylogenetic analysis clustered the PyCCR*s* into two distinct clades: PyCCR*1~6* fall into the CCR clade, while the remaining members form a CCR-like clade. In this study, twelve ORF regions of the *P. yunnanensis CCR* genes were cloned, and nine purified recombinant PyCCR proteins were obtained through prokaryotic expression. In vitro enzymatic assays demonstrated that PyCCR1, PyCCR2, PyCCR5, and PyCCR6 catalyzed the conversion of *p*-coumaroyl-CoA, feruloyl-CoA, and sinapoyl-CoA to *p*-coumaraldehyde, coniferaldehyde, and sinapaldehyde, respectively. Molecular docking of PyCCR1 to 6 with three substrates identified substrate-binding pocket domains. Within these domains, hydrogen bonds formed between ligands and residues in the R(X)5K motif of PyCCR1 to 6, whereas PyCCRL7 to 12 lacked the complete motif. RT-qPCR analysis showed tissue-specific expression patterns of the 12 genes across buds, stems, leaves, roots, and fruits. Collectively, these findings suggest that the presence of a complete R(X)5K motif may play a crucial role in maintaining the catalytic activity of PyCCRs. Our present study established a mechanistic foundation for elucidating lignin biosynthesis regulation in *P. yunnanensis* and offer genetic resources for improvement programs.

## 1. Introduction

Lignin represents the second most abundant terrestrial biopolymer, with an estimated annual biosynthesis rate of approximately 200 billion metric tons globally [1]. This biopolymer fulfills essential physiological functions in vascular plants, predominantly depositing in secondary cell walls [2], where it confers mechanical strength, maintains vascular integrity, and enhances resistance to diverse biotic and abiotic stresses [3,4]. Although lignin polymers exhibit remarkable structural complexity in plants, their primary constituents derive from the oxidative coupling of three monolignols: *p*-coumaryl alcohol (H-unit), coniferyl alcohol (G-unit), and sinapyl alcohol (S-unit) [5]. Generally, ferns and gymnosperms accumulate lignin predominantly containing G units, supplemented by minor levels of H units. By contrast, angiosperm lignins predominantly comprise G and S units, with H units present as minor constituents. Notably, dicotyledonous angiosperms typically incorporate both G and S units, whereas monocotyledons exhibit comparable proportions of all three monolignols [4,6].

Lignin biosynthesis proceeds through three interconnected pathways. First, the shikimate pathway synthesizes phenylalanine, tyrosine (predominantly in graminaceous species), and tryptophan from photosynthetically derived glucose; Next, The phenylpropanoid pathway converts phenylalanine to cinnamoyl-CoA esters via cinnamic acid; Finally, The lignin-specific pathway reduces cinnamoyl-CoA esters via cinnamyl alcohol dehydrogenase (CAD) to yield monolignols, which undergo oxidative polymerization forming lignin [7,8]. The phenylpropanoid pathway facilitates lignin monomer synthesis through enzymatic catalysis. Lignin polymers are derived from three monolignol classes: H), G, and S units. The H-unit biosynthesis involves: *p*-coumaric acid → *p*-coumaroyl-CoA → *p*-coumaraldehyde → *p*-coumaryl alcohol. The G-unit pathway proceeds through: ferulic acid → feruloyl-CoA → coniferaldehyde → coniferyl alcohol. The S-unit pathway follows: sinapic acid → sinapoyl-CoA → sinapaldehyde → sinapyl alcohol [9,10]. Cinnamoyl-CoA reductase (CCR, EC 1.2.1.44) serves as the key rate-limiting enzyme, catalyzing cinnamoyl-CoA conversion to cinnamaldehyde and thus regulating carbon flux into lignin biosynthesis.

As the first committed enzyme in the lignin biosynthetic branch, CCR catalyzes the irreversible conversion of cinnamoyl-CoA to cinnamaldehydes [11]. CCR exhibits broad substrate specificity, catalyzing *p*-coumaroyl-CoA, feruloyl-CoA, and sinapoyl-CoA into *p*-coumaraldehyde, coniferaldehyde, and sinapaldehyde, respectively—confirming its role as the pivotal rate-limiting enzyme [12]. These aldehydes serve as the immediate precursors for monolignol synthesis, which are subsequently reduced to *p*-coumaryl, coniferyl, and sinapyl alcohols to undergo lignin polymerization. The catalytic profiles yielding specific aldehydes vary across plant species, reflecting the phylogenetic divergence that ultimately shapes downstream lignin composition. These hydroxycinnamyl aldehydes are substrates for CAD-mediated reduction. CCR directly modulates the abundance and composition of lignin monomers (*p*-coumaryl, coniferyl, and sinapyl alcohols) in plant. CCR functions as a metabolic control node, regulating carbon flux partitioning into lignin biosynthesis and overall deposition rates. As the gateway enzyme, CCR coordinates downstream enzymes to precisely regulate lignin biosynthesis. This strategic role establishes CCR as a prime target for modulating lignin properties in genetic engineering [11,13,14].

Mansell et al. (1972) first elucidated the molecular mechanisms of CCR-mediated lignin biosynthesis, establishing the enzymatic basis for monolignol formation [15]. Goffner et al. (1994) achieved a landmark breakthrough by cloning and characterizing the first CCR gene from *Eucalyptus gunnii* (Hook.f.) [16]. The conserved KNWYCYGK motif is widely documented in the CCR gene family. The family comprises two distinct subclasses: bona fide CCRs, which possess definitive catalytic activity toward monolignol biosynthesis intermediates, and CCR-like genes, which often display altered substrate-binding motifs and are hypothesized to participate in diverse, non-lignin specialized metabolic pathways or defense responses [17,18,19,20,21]. However, this motif serves solely for family-level identification, lacking specificity for distinguishing CCR from CCR-like subclasses. CCR has been extensively characterized across plant taxa, with significant interspecific variation in gene family size. Current CCR research predominantly focuses on angiosperms. Comparative genomics reveals substantial CCR family size variation in angiosperms: 11 in *Arabidopsis thaliana* [22], 115 in *Triticum aestivum* [23], 9 in *Populus trichocarpa* [24], 33 in *Oryza sativa* [25], and 22 in *Linum usitatissimum* [20]. The functional characterization of *CCR* genes has been extensively documented across diverse angiosperm lineages. In *A. thaliana*, loss-of-function mutations in AtCCR1 significantly alter ferulic acid (FeA) accumulation, leading to impaired leaf cell proliferation phenotypes [26]. In *Morus alba*, MaCCR1 can catalyze feruloyl-CoA and sinapoyl-CoA independently. After knocking out *MaCCR1*, it was found that the lignin content in the stems and leaves was significantly reduced [21]. *Panicum virgatum* PvCCR-1/2 exhibit catalytic activity toward five substrates: *p*-coumaroyl-CoA, caffeoyl-CoA, feruloyl-CoA, 5-hydroxyferuloyl-CoA, and sinapoyl-CoA, with distinct substrate preferences [27]. In contrast, CCR roles in gymnosperm lignin biosynthesis remain understudied, with characterization limited to few conifers, such as *Picea abies* [28] and *Pinus radiata* [29].

*P. yunnanensis* (Pinaceae), an ecologically and economically important gymnosperm, exhibits drought-resistant adaptations and serves as a major timber species. Endemic to the Yungui Plateau and southwestern China, *P. yunnanensis* occupies 23–30° N, 96–108° E at 400–3100 m elevation. *P. yunnanensis* holds significant economic value as premium construction timber, industrial raw material, and the dominant pulpwood resource in this region. Coniferous species retain higher pulp lignin content than broadleaves [30]. Elevated pulp lignin content increases chemical delignification costs, hazardous emissions, and environmental pollution [31]. CCR, the first committed enzyme of the monolignol branch, regulates metabolic flux toward lignin biosynthesis. Thus, elucidating CCR regulation in *P. yunnanensis* lignin biosynthesis and applying genetic engineering to modulate lignin content would facilitate improved varieties and sustainable production.

To discriminate authentic CCR genes in *P. yunnanensis*, we performed phylogenetic analysis using the following sequences: reference CCR and CCR-like sequences with experimentally validated functions, and the CCR gene repertoire identified from the transcriptome of *P. yunnanensis.* This approach enabled clear differentiation between CCR and CCR-like genes in this conifer species. Here, we present the first transcriptome-wide systematic analysis of the CCR gene family in *P. yunnanensis* and experimentally validates its catalytic function using enzyme activity assays.

## 2. Materials and Methods

### 2.1. Plant Materials and Reagents

*P. yunnanensis* samples were collected on 1 March 2023 from the nursery of the college of forestry, Southwest Forestry University (N 25°04′00″, E 102°45′41″, altitude 1945 m) as experimental materials. The samples consisted of one-year-old *P. yunnanensis* seedlings that were growing well and free of pests and diseases. Additionally, to ensure a comprehensive tissue expression profile, developing cones (referred to as fruits in this study) were independently collected from mature *P. yunnanensis* trees growing in the same geographical region. Fast Pure^®^ Universal Plant Total RNA Isolation Kit, Hi Script^®^ III 1st Strand cDNA Synthesis Kit (+gDNA wiper), and Taq Pro Universal SYBR qPCR Master Mix were purchased from Vazyme Biotech Co., Ltd. (Nanjing, China). The M-MLV 4 First Strand cDNA Synthesis Kit (Biomed, Shanghai, China).

### 2.2. Identification and Characterization of CCR Genes and Proteins in P. yunnanensis

The *P. yunnanensis* transcriptome dataset utilized in this study was generated from nine RNA-seq samples, comprising tender stems, buds, and needles collected from one-year-old *P. yunnanensis* seedlings (three biological replicates for each tissue type). To eliminate redundancy caused by alternative splicing and sequencing artifacts inherent in the assembly process, the transcripts were subjected to a clustering step, where only the longest representative transcript from each cluster was extracted to form a non-redundant Unigene library. Consequently, our identification procedures were conducted exclusively on these unigenes. The identification and screening of the *PyCCR* gene family members were conducted based on a transcriptome dataset obtained from our previously published study [32]. We searched Swiss-Prot using “cinnamoyl-CoA reductase” and “CCR” as query terms and retained sequence IDs annotated with these functions. Meanwhile, we constructed a Hidden Markov Model (HMM) for cinnamyl-CoA reductase using HMMER, performed BLASTp analysis against the *P. yunnanensis* transcriptome protein sequences with an E-value cutoff of 0.001, and identified candidate CCR sequences in *P. yunnanensis*. The accession numbers of the candidate CCR genes were then used to extract the corresponding CDS sequence from the *P. yunnanensis* transcriptome data. Then, the candidate genes obtained by the above two methods were validated using the NCBI Conserved Domain Database (https://www.ncbi.nlm.nih.gov/Structure/cdd/wrpsb.cgi, accessed on 20 July 2023) to confirm conserved domains, followed by removal of redundant sequences. The screening results were integrated, yielding standardized gene fragments representing *PyCCRs* genes. The physicochemical properties of PyCCR*s* were determined using ExPASy tools, including relative molecular mass, isoelectric point (pI), and the sums of Arg + Lys and Asp + Glu residues.

### 2.3. Total RNA Extraction, RNA Reverse Transcription, and cDNA Cloning

Total RNA was extracted from one-year-old *P. yunnanensis* seedlings using the FastPure^®^ Universal Plant Total RNA Isolation Kit as recommended by the manufacturer. RNA quality was assessed by measuring absorbance using a Nano-500 micro-spectrophotometer (Allsheng, Hangzhou, China) and by agarose gel electrophoresis. cDNA was synthesized from RNA using the M-MLV 4 First Strand cDNA Synthesis Kit (Biomed, Shanghai, China) and stored at −20 °C for subsequent use. Composition of the reverse transcription reaction is presented in Table S1. The reaction was incubated at 37 °C for 30 min in a Touch thermal cycler (Heal Force, Shanghai, China), then terminated at 85 °C for 10 min.

### 2.4. Multiple Sequence Alignment and Phylogenetic Analysis of CCRs from P. yunnanensis

PyCCR*s* amino acid sequences were analyzed by BLASTP against the NCBI non-redundant protein database to identify homologous cinnamoyl-CoA reductase sequences. Multiple sequence alignment was performed with DNAMAN 8.0, followed by phylogenetic tree construction in MEGA 11.0 using the Maximum Likelihood algorithm (LG + G model, 1000 bootstrap replicates) In addition to functionally characterized CCR and CCR-like sequences from model angiosperms (*A. thaliana*, *P. tremuloides*, etc.), representative CCR candidates from diverse gymnosperms were retrieved from the NCBI database, including conifers (*P. taeda* and *P. massoniana*) and *Ginkgo biloba*. Phylogenetic trees were visualized using the Circle Tree web tool (https://www.chiplot.online/circleTree.html, accessed on 8 May 2025).

### 2.5. Cloning and Homologous Recombination of CCR Candidate Genes in P. yunnanensis

The coding sequences (CDS) of 12 putative cinnamoyl-CoA reductase genes (*PyCCR1~PyCCR12*) were analyzed using NCBI ORF Finder (https://www.ncbi.nlm.nih.gov/orffinder/, accessed on 21 July 2023) to identify complete open reading frames (ORFs), which yielded full-length ORF sequences for all CCR family members in *P. yunnanensis*. Primers containing BamHI (GGATCC) and SalI (GTCGAC) restriction sites were designed against the pET30a (+) multiple cloning site (MCS) sequence using SnapGene 6.0 (Insightful Science; https://www.snapgene.com/) to amplify *PyCCR1~PyCCR12* genes (Table S2). All primers were commercially synthesized by Sangon Biotech (Shanghai, China).

Cloning was performed using the CloneExpress II One-Step Cloning Kit (Vazyme, Nanjing, China) according to the manufacturer’s protocol. RT-PCR amplification was performed using 2 × Phanta Max Master Mix (Dye Plus; Vazyme, Nanjing, China) following the manufacturer’s protocol (Figure S1). The reaction system composition and thermal cycling conditions are provided in Tables S3 and S4, respectively. The pET30a (+) plasmid was digested with the corresponding restriction enzyme using the reaction system specified in Table S5. The digested product was purified and used for subsequent cloning with the CloneExpress II One-Step Cloning Kit (Vazyme, China) following the manufacturer’s instructions. The homologous recombination reaction was assembled as described in Table S5. After 30 min incubation at 37 °C, the reaction mixture was used directly for transformation.

DH5α competent cells (Takara, Tokyo, Japan) were thawed on ice and maintained at −80 °C in an ultra-low temperature freezer. The pET30a (+) plasmid was mixed with competent cells (1:10 ratio) by gentle pipetting and incubated on ice for 30 min.

Heat shock was performed at 42 °C for 45 s in a metal bath, followed by immediate ice incubation for 2–3 min. Cells were resuspended in 400 μL antibiotic-free LB medium and incubated at 37 °C with 180 rpm shaking for 1 h. Samples were centrifuged at 5000× *g* for 5 min using a high-speed centrifuge. Under sterile conditions, the supernatant was discarded and cells were resuspended in the 50 μL of remaining medium, plated on kanamycin-containing LB agar, spread with a sterile spreader, sealed with parafilm, and incubated at 37 °C for 12–16 h. Positive clones were verified by PCR (Figure S2), with each gene amplified in triplicate. Sanger sequencing was performed by Sangon Biotech (Shanghai, China).

### 2.6. Heterologous Expression and Purification of PyCCRs Proteins

Following successful cloning and sequencing of *PyCCR1~PyCCR12* genes from *P. yunnanensis*, the recombinant pET30a (+)-*PyCCR1~PyCCR2* plasmids were purified and transformed into *Escherichia coli* BL21(DE3) competent cells.

To optimize protein induction conditions, we examined IPTG concentration, induction temperature, and induction time. By setting different IPTG concentrations and temperature gradients, we obtained the optimal induction concentration and temperature. Protein expression was induced in the recombinant *E. coli* BL21(DE3) strains at mid-log phase (OD_600_ = 0.6–0.8) with 0.2 mM isopropyl-β-D-1-thiogalactoside (IPTG). The cultures expressing PyCCR1, PyCCR2, PyCCR3, PyCCR5, PyCCR6, PyCCRL7, PyCCRL9, and PyCCRL12 were then incubated at 28 °C for 10 h, while those expressing PyCCR4, PyCCRL8, PyCCRL10, and PyCCRL11 were incubated at 16 °C for 10 h, both with shaking at 180 rpm. All recombinant proteins were obtained carrying a 6×His tag (Figure S4). To establish optimal elution conditions, soluble recombinant proteins from induced cultures were purified by nickel-affinity chromatography. The PyCCRs-6×His fusion protein was eluted with imidazole step gradients (40, 80, 150, and 250 mM) in elution buffer.

### 2.7. In Vitro Enzymatic Activity Assay of PyCCRs

The concentration of *P. yunnanensis* CCRs recombinant protein was determined using the Bradford method, with reference to the instructions for use of the Detergent Compatible Bradford Protein Quantification Kit (Vazyme Biotech Co., Ltd., Nanjing, China) (Table S6).

To investigate the lignin-catalyzing capacity of PyCCR proteins, purified recombinant PyCCRs were analyzed using an in vitro enzymatic assay. Substrates were selected based on their involvement in the lignin biosynthesis pathway, including feruloyl-CoA, sinapoyl-CoA and *p*-coumaroyl-CoA.

To evaluate substrate specificity, independent single-substrate reactions were performed. Each 500 µL reaction system contained 0.15 mM of either *p*-coumaroyl-CoA, feruloyl-CoA, or sinapoyl-CoA as the sole substrate, along with 0.16 mM NADPH, 32 μg of PyCCRs-6×His, and phosphate saline buffer (PBS, pH 6.25). Reactions were conducted at 35 °C for 30 min and terminated by boiling (100 °C, 10 min). Products were analyzed by HPLC (Agilent 1260 Infinity II) equipped with a ZORBAX SB-C18 column (150 × 4.6 mm, 5 μm) using a 10 μL injection volume (Table S7). Absorbance was monitored at 280, 290, 308, and 333 nm. Triplicate experiments were performed, with data analysis conducted using Origin 2024. Heat-inactivated recombinant PyCCR proteins (boiled at 100 °C for 10 min) were employed as negative controls to ensure that the observed reduction was exclusively enzymatically driven.

### 2.8. Homology Modelling and Molecular Docking of PyCCRs from P. yunnanensis

Protein structures of PyCCR1~6 were predicted using AlphaFold2. The protein sequences of PyCCR1~6 in *P. yunnanensis* show the following homology in the AlphaFold2 model predictions: PyCCR1 has 100% homology with the CCR (AF-A0A076NB75-F1-v4) of *P. koraiensis*; PyCCR2 and PyCCR3 share 85% and 84% homology, respectively, with the CCR (AF-A0A0G7ZNS3-F1-v4) of *P. glauca.* PyCCR4 shares 89% homology with the CCR (AF-A0A0G7ZNX3-F1-v4) of *P. glauca.* PyCCR5 shares 92% homology with the CCR (AF-A0A0G7ZNT0-F1-v4) from *P. glauca*, PyCCR6 shares 87% homology with the CCR (AF-A0A0G7ZP15-F1-v4) from *P. glauca*, and PyCCRL7 shares 94% homology with the CCR (AF-A9NL44-F1-v4) of *P. sitchensis*. Small molecule 2D structures were obtained from PubChem and converted to 3D models using Chem3D 15.0 (ChemOffice 2015). Energy minimization was performed using the MMFF94 force field to optimize molecular geometries. Molecular docking was conducted with AutoDock 4.2.6 using default parameters. Docking results were visualized and analyzed with PyMOL 2.6.0.

### 2.9. RT-qPCR Analysis of the CCR Genes in P. yunnanensis

Total RNA was extracted from five tissues (buds, stems, leaves, and roots from seedlings, alongside fruits from mature trees) of *P. yunnanensis* using the FastPure^®^ Universal Plant Total RNA Isolation Kit (Vazyme, Nanjing, China) following the manufacturer’s protocol. Three biological replicates were prepared for each sample. Following the HiScript^®^ IV RT SuperMix for qPCR cDNA (+gDNA wiper) (Vazyme, China) protocol, qualified RNA samples were reverse-transcribed into cDNA using tissue-specific RNA templates and stored at −20 °C in a medical freezer for subsequent analysis.

RT-qPCR primers were designed using *P. yunnanensis* cDNA as a template, with *Actin-4* as the reference gene across different tissues. Total RNA was extracted from three biological replicates per tissue, and cDNA was synthesized. The quality of cDNA templates was verified by both agarose gel electrophoresis (to confirm integrity) and spectrophotometric measurement (A260/A280 ratio between 1.8 and 2.0). RT-qPCR was performed using the 2 × Taq Pro Universal SYBR qPCR Master Mix according to the manufacturer’s protocol. The specificity of amplification was verified by melting curve analysis. Each reaction was run in three technical replicates. The relative expression levels of CCR gene family members were calculated using the 2−ΔΔCt method. Statistical analysis was performed using GraphPad Prism (v.9.0) (significance threshold of *p* < 0.05). The RT-qPCR reaction conditions are detailed in Tables S8–S10.

## 3. Results

### 3.1. Identification of CCR Genes in P. yunnanensis

To systematically identify the CCR gene family in *P. yunnanensis*, a transcriptome-wide workflow integrating keyword screening, local BLASTp, and HMMER-based hidden Markov model (HMM) profile searches was executed against the *P. yunnanensis* transcriptome database, targeting the characteristically conserved NAD(P)-binding motif (KNWYCYGK). These results were compiled into annotated consensus gene fragments of cinnamoyl-CoA reductase, with 12 CCR family members identified in *P. yunnanensis*. These genes are named *PyCCR1* to *PyCCRL12* according to their gene ID numbers (Table 1). The open reading frame (ORF) lengths of the identified *PyCCR* genes ranged from 795 to 1074 bp, encoding proteins comprising 264 to 357 amino acid residues, with an average length of 324 amino acids. The predicted molecular weights of these proteins ranged from 29.69 to 39.82 kDa, while their predicted isoelectric points (pI) ranged from 5.13 to 8.22 (mean: 6.38). Subcellular localization prediction suggested that the CCR family members in *P. yunnanensis* predominantly reside in the cytoplasm, nucleus, and mitochondria (Table S11).

### 3.2. Phylogenetic Analysis of P. yunnanensis CCRs

**Figure 1:**
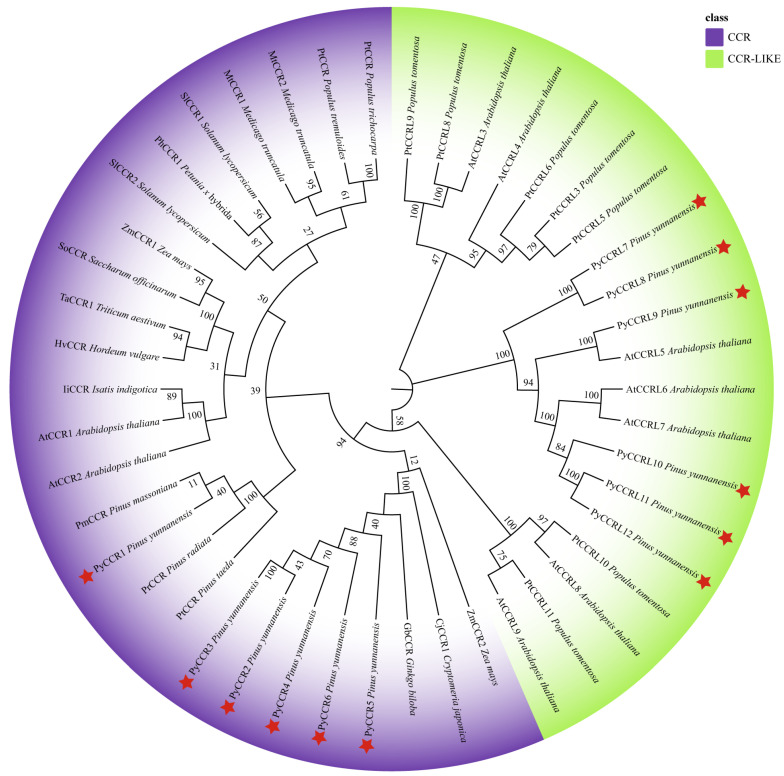
Phylogenetic tree of *P. yunnanensis* CCRs and other plant CCRs. The purple background indicates true CCR branches, while the cyan background indicates branches similar to CCR-like. The red star indicates the CCR of *P. yunnanensis.*

To elucidate the evolutionary relationships and line-specific divergence of the PyCCR family, a Maximum Likelihood (ML) phylogenetic tree was constructed using full-length protein sequences of verified and putative CCR and CCR-like proteins from 15 representative plant taxa across angiosperms and gymnosperms (Figure 1). Furthermore, to resolve gymnosperm-specific evolutionary trajectories, putative CCR homologs from *P. taeda*, *P. massoniana*, and *G. biloba* were integrated into the dataset. The resulting phylogenetic topology reveals that the 12 candidates partitioned into two highly robust monophyletic lineages: PyCCR1–PyCCR6 tightly clustered within the real CCR clade alongside functional gymnosperm orthologs, while the remaining members (PyCCRL7–PyCCRL12) branched exclusively into the CCR-like clade.

### 3.3. Conserved Structural Domains of Proteins Encoded by CCR Genes in P. yunnanensis

**Figure 2:**
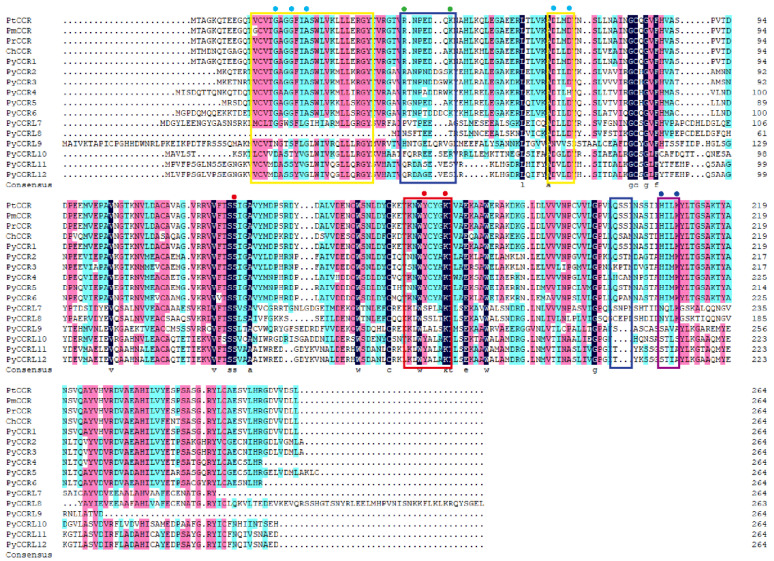
Alignment of *P. yunnanensis* CCR protein. Cyan dots indicate G(X)2G(X)2 A/G and D(X)2D motifs belonging to the NAD(P) binding motif. Green dots indicate R(X)5 K motifs involved in NADPH binding. Red dots indicate the catalytic triad SYK. Blue dots indicate H(X)2K motifs. The red boxes highlight the core conserved motifs, whereas other colored boxes indicate the positions of respective characteristic motifs.

To further resolve the domain architecture and sequence conservation of the identified candidates, multiple sequence alignment of PyCCR1–6 and PyCCRL7–12 was performed alongside representative gymnosperm and conifer orthologs (including *P. radiata*, *P. massoniana*, *P. taeda*, and *Cephalotaxus hainanensis*) (Figure 2). Protein sequence analysis revealed that all *PyCCRs* except *PyCCRL8*, possessed the hallmark features of the short-chain dehydrogenase/reductase (SDR) superfamily: the Ser-Tyr-Lys (SYK) catalytic triad and the Rossmann fold domain for NAD(P) binding. Furthermore, the core motifs G-X-X-G-X-X-A and D-X-X-D, which coordinately facilitate NAD(P) binding and stabilize the adenine-binding pocket [33], were strictly conserved in all candidates except PyCCRL8 and PyCCRL9. Notably, the NADP+-specific motifs, R(X)5K, R(X)6K, and R(X)9K, were exclusively identified in the bona fide CCR clade (PyCCR1–6), which robustly differentiates authentic CCRs from other NAD(H)-dependent SDR family members [11,34,35]. Specifically, PyCCR1 and PyCCR5 harbor the canonical R(X)5K motif, whereas PyCCR2–4 and PyCCR6 exhibit expanded spatial variants (R(X)6K and R(X)9K) that may modulate NADPH-binding affinity. Consistent with previous studies highlighting the indispensable role of the CCR signature motif (NWYCYGK) in catalytic function, our alignment confirmed that PyCCR1 and PyCCR5 retain the intact NWYCYGK motif. Conversely, PyCCR2–4 and PyCCR6 display a single amino acid substitution (Y → A) at the sixth position, while the CCR-like members (PyCCRL7–12) lack this essential domain. Moreover, the highly conserved H(X)2K motif (representing the CCR-specific substrate-binding motif, CCR-SBM) was exclusively retained in PyCCR1–6, which is an absolute prerequisite for CCR enzymatic activity [18]. Collectively, these distinct sequence signatures provide compelling molecular evidence for classifying PyCCR1–6 as bona fide CCRs, while distinguishing the structurally divergent PyCCRL7–12 as CCR-like proteins.

### 3.4. Expression of Recombinant Proteins of P. yunnanensis CCRs

The 6×His-tagged recombinant proteins PyCCR1 through PyCCRL12 were purified by nickel-nitrilotriacetic acid (Ni-NTA) affinity chromatography using an imidazole gradient in lysis buffer. Soluble proteins were successfully purified for nine of the twelve PyCCRs (PyCCR1, PyCCR2, PyCCR5, PyCCR6, PyCCRL7, PyCCRL8, PyCCRL9, PyCCRL10, and PyCCRL12), whereas PyCCR3, PyCCR4, and PyCCRL11 were not obtained in soluble form. The purified proteins were characterized using sodium dodecyl sulfate–polyacrylamide gel electrophoresis (SDS-PAGE), and their concentrations are shown in Figures S5 and S6.

### 3.5. Functional Characterisation of Recombinant P. yunnanensis CCR Proteins

**Figure 3:**
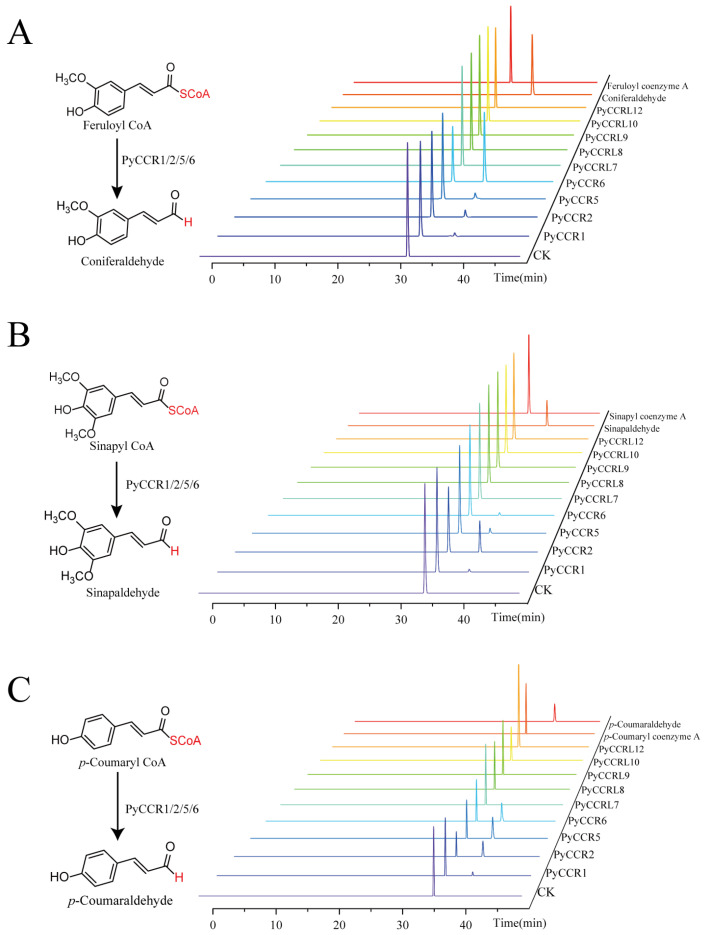
In vitro enzymatic activity analysis of recombinant PyCCR proteins from *P. yunnanensis*. (**A**) HPLC chromatograms of reaction products demonstrating the catalytic activity of PyCCR1, PyCCR2, PyCCR5, PyCCR6, PyCCRL7, PyCCRL9, PyCCRL10, PyCCRL11, and PyCCRL12 toward feruloyl-CoA. (**B**) Enzymatic assays using sinapoyl-CoA as a substrate. (**C**) Enzymatic assays using *p*-coumaroyl-CoA as a substrate. Red text indicates the specific peaks or chemical sites of interest.

To investigate the lignin-catalyzing capacity of PyCCR proteins, we focused our enzymatic assays on the nine soluble members obtained. HPLC analysis demonstrated that PyCCRs exhibited reductase activity toward feruloyl-CoA, sinapoyl-CoA, and *p*-coumaroyl-CoA (Figure 3). HPLC analysis showed a characteristic peak with a retention time (Rt = 38.025 min) consistent with that of the coniferaldehyde standard (Rt = 38.572 min). The comparable retention times suggested identification of the peak as coniferaldehyde (Figure 3A). When sinapoyl-CoA served as substrate, PyCCRs demonstrated catalytic activity toward sinapoyl-CoA conversion (Figure 3B). The reaction mixtures contained a sinapaldehyde peak (Rt = 40.421 min), while the standard eluted at 40.949 min. Chromatographic comparison with the standard suggested the new peak corresponded to sinapaldehyde, based on retention time matching. When *p*-coumaroyl-CoA was used as a substrate, PyCCRs exhibited catalytic activity in *p*-coumaroyl-CoA conversion. HPLC analysis of the reaction products revealed a *p*-coumaraldehyde peak (Rt = 38.901 min). The *p*-coumaraldehyde reference standard eluted at 39.063 min. The retention time alignment indicated successful *p*-coumaraldehyde production (Figure 3C).

### 3.6. Homology Modelling and Molecular Docking of CCRs from P. yunnanensis

**Figure 4:**
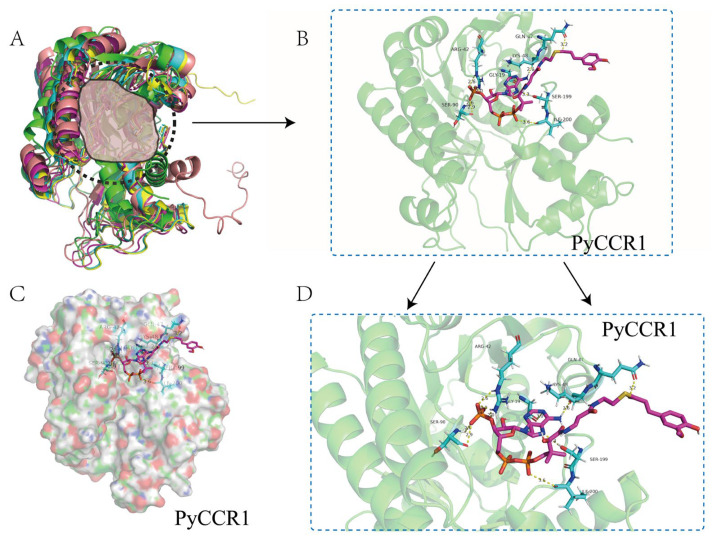
Molecular docking of PyCCR1 with feruloyl coenzyme A. (**A**) Three-dimensional crystal cartoon models of PyCCR1~6. (**B**) Binding site of PyCCR1 with feruloyl-CoA. The feruloyl-CoA substrate (pink) is represented as a stick model. Residues are shown as stick models with highlighted markers (cyan). Hydrogen bonds are displayed as sticks (yellow). (**C**) Surface structure of PyCCR1 bound to the substrate. (**D**) Interactions between feruloyl-CoA (stick model) and the catalytic active site residues GLY-19, GLN-47, SER-90, ARG42, and SER-199 (ball-and-stick models) of PyCCR1, forming a pocket structure.

Comparative structural analysis of the models (Figure 4A) was conducted, followed by 50 independent genetic algorithm-based docking simulations to determine the optimal enzyme–substrate conformation. Docking results demonstrated that *p*-coumaroyl-CoA, feruloyl-CoA, and sinapoyl-CoA were all positioned within the catalytic binding pocket of PyCCR1~6. The substrate-binding pocket is delineated by multiple residues, including GLY-17, THR-40, ARG-42, LYS-48, GLN-47, GLU-98, SER-199, and THR-205, which collectively constitute a substrate-access channel. Molecular docking analysis revealed hydrogen bond formation between the substrates and both R(X)5K (including R(X)6K and R(X)9K variants) as well as catalytic site residues (Figure 4B–D). Structural analysis demonstrated that in most PyCCRs, substrates establish multiple hydrogen bonds with ARG-42 (corresponding to the R residue in the R(X)5K motif) (Figures S6–S8), indicating its potential key role in the catalytic site of CCRs. To investigate the interaction, we conducted molecular docking of PyCCRL7 with its substrate, feruloyl-CoA. The results indicated that the absence of the key R(X)5K motif in PyCCRL7 results in an incomplete pocket domain, thereby preventing the ligand from binding to key residues. These findings demonstrate that the R(X)5K structural motif is crucial for substrate binding. Previous studies reported docking analysis between a *Leucaena leucocephala* CCR homology model and feruloyl-CoA. ARG51 was identified as a critical catalytic residue in *Leucaena leucocephala* CCR, demonstrating multiple hydrogen bond interactions with the substrate [36]. It belongs to the R(X)5K structure, as does ARG-42 in *P. yunnanensis*. Therefore, we speculate that *P. yunnanensis* PyCCRL7~12 may lack the R(X)5K structure, resulting in the absence of catalytic activity in PyCCRL7~12 and affecting substrate binding.

### 3.7. RT-qPCR Analysis of CCRs Gene Expression Levels in P. yunnanensis

**Figure 5:**
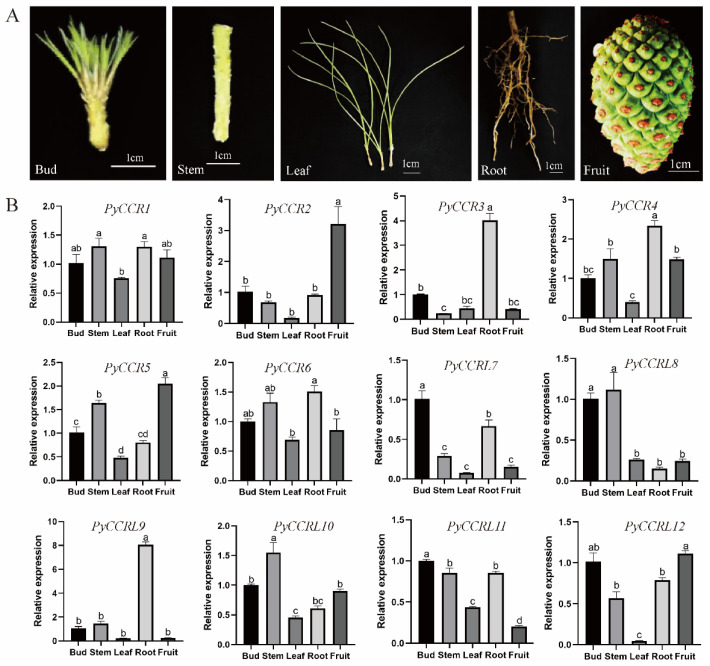
Relative expression of *PyCCRs* gene in different tissues of *P. yunnanensis.* (**A**) Morphological observations of the five representative tissues (bud, stem, leaf, root, and fruit) used for expression analysis. Scale bars = 1 cm. (**B**) Tissue-specific expression profiles of the 12 *PyCCR* family members determined by RT-qPCR. Data are presented as the mean ± standard deviation (SD) of three biological replicates. Different lowercase letters above the bars indicate significant differences among different tissues (*p* < 0.05).

To validate the tissue-specific and developmental stage-dependent expression profiles of *PyCCR* genes, RT-qPCR was employed to analyze the transcript levels of CCR gene family members (Figure 5). The results indicated that transcripts of all *PyCCR* genes in *P. yunnanensis* were detectable in all five tissues examined, with *PyCCR1*, *PyCCR4*, *PyCCR5, PyCCR6*, *PyCCRL8*, *PyCCRL9*, and *PyCCRL10* showing primary expression in the stem; *PyCCR2*, *PyCCR3*, *PyCCRL7*, *PyCCRL11*, and *PyCCRL12* showed high-level expression in buds. It was also found that *PyCCR3*, *PyCCR4*, and *PyCCRL9* were strongly expressed in the roots. Integrated analysis of the tissue-specific expression patterns (RT-qPCR) and the catalytic activities of recombinant proteins (HPLC) indicated that PyCCR1/4/5/6 and PyCCRL8/9/10 are likely responsible for cinnamaldehyde production in stems, while PyCCR2/3 and PyCCRL7/11/12 may mediate this conversion in buds. Although the *CCR-like* (*PyCCRL*) members are highly expressed in specific tissues, their lack of in vitro catalytic activity implies they might be involved in other unknown biological processes rather than direct lignin monomer biosynthesis.

## 4. Discussion

### 4.1. Identification of the CCR Gene Family in P. yunnanensis

Plant metabolic profiles exhibit complexity, characterized by both primary and secondary metabolites [37,38]. The phenylpropanoid pathway is responsible for the synthesis of one of the three major classes of plant secondary metabolites (phenolics). Through these pathways, plants directly or indirectly synthesize substances containing a phenylpropanoid skeleton, such as lignin, flavonoids, and anthocyanins [39,40,41]. Lignin is a complex organic polymer composed of three major monolignols [5] and is a key component of plant cell walls. It is essential for maintaining mechanical rigidity during plant development, maintaining vascular integrity for water transport [26], and reinforcing defense mechanisms against environmental stresses, including drought.

The CCR gene family displays ubiquitous distribution across plant taxa while exhibiting significant sequence and functional diversity among species. Phylogenetic analyses have characterized CCR family members in diverse plant species, including *A. thaliana*, *Lolium perenne*, *P. trichocarpa*, *P. tomentosa*, *O. sativa*, and *Malus domestica*. Despite the gene family’s extensive size, only a subset of CCRs demonstrates catalytic activity toward lignin pathway intermediates, with most members classified as CCR-like proteins [35]. To date, a complete genome of *P. yunnanensis* has not been reported, and the transcriptome data utilized in this study represents only a portion of the transcriptional information. Because a complete genome for *P. yunnanensis* is not yet available, our identification relies entirely on transcriptome datasets. Consequently, this study may not encapsulate the absolute complete *PyCCR* gene family. Lowly expressed, stress-inducible, or highly tissue-specific *PyCCR* genes might be absent from our current RNA-seq libraries. Furthermore, despite rigorous deduplication, we cannot completely rule out the possibility that highly similar transcripts represent alternative splicing events rather than distinct paralogous genes. Future availability of a high-quality chromosome-level genome will be required to definitively resolve the absolute size and genomic organization of the *PyCCR* family. In this study, we identified 12 CCR family genes in *P. yunnanensis*. To distinguish functional CCR genes, we performed phylogenetic analysis by aligning the 12 PyCCR sequences with known CCR and CCR-like genes from other species. Phylogenetic clustering placed PyCCR*1* through PyCCR*6* within the conserved CCR clade.

Previous studies have established that the conserved CCR motif NWYCY and the NADP-binding motif R(X)5K serve as key diagnostic features for distinguishing CCR and CCR-like enzymes from other NAD(H)-dependent short-chain dehydrogenases/reductases (SDRs) [33]. We identified twelve *PyCCR* genes in *P. yunnanensis*, with PyCCR*1* through PyCCR*6* containing the conserved NWYCYGK catalytic motif characteristic of functional CCR enzymes. Notably, PyCCR*2* to PyCCR*6* exhibit an alanine substitution at the sixth position (G → A), a variation frequently observed in CCR families across plant species. Furthermore, PyCCR*1*~*6* all contain the R(X)5K structural motif that differentiates CCRs from NAD(H)-dependent short-chain dehydrogenases/reductases [34]. Structural analysis of the CCRs and CCR-like proteins, combined with multiple sequence alignment, revealed that the R(X)5K structural motif is located within the substrate-binding pocket domain. Molecular docking experiments demonstrated that the R(X)5K motif forms multiple hydrogen bonds with the substrate ligands. In contrast, PyCCRLs appear to lack this motif, which results in their inability to catalyze substrates. Thus, it is proposed that these key residues act as rigid structural elements in PyCCRs, thereby facilitating ligand coordination and binding. Furthermore, the Q(X)2 motif in PyCCRs (except PyCCR3) was observed to form hydrogen bonds with the substrates. However, multiple sequence alignment revealed the absence of the Q(X)2 motif in both PyCCRLs and PyCCR3. Based on these findings, it is hypothesized that the R(X)5K and Q(X)2 motifs within the pocket domain collectively form a robust hydrogen bond network that securely positions the substrate for catalysis. The conserved Ser-Tyr-Lys (SYK) catalytic triad, characteristic of short-chain dehydrogenases/reductases (SDRs), has been demonstrated to be essential for enzymatic activity [42]. Among the PyCCR family members, PyCCR*1~6* and PyCCR*L10~12* contain intact Ser-Tyr-Lys catalytic triads, whereas PyCCR*L7* to PyCCR*L9* exhibit tyrosine residue deletions that likely impair NADPH binding and abolish CCR enzymatic function. Additionally, PyCCR*1* through PyCCR*6* contain the conserved H(X)2K motif (CCR-specific substrate-binding motif, CCR-SBM) [18], which serves as a molecular signature to differentiate functional CCRs from CCR-like proteins and is essential for catalytic activity.

### 4.2. Biochemical Properties of PyCCRs

Extensive characterization of CCR enzymatic properties and biological functions across diverse plant species has revealed species-specific variations in lignin composition [16,27,33]. Gymnosperm lignins predominantly consist of guaiacyl (G) units derived from coniferyl alcohol. In contrast, angiosperm lignins are typically composed of both guaiacyl (G) and syringyl (S) units, which are polymerized from coniferyl and sinapyl alcohols, respectively. The relative abundance of *p*-hydroxyphenyl (H) units shows considerable variation both interspecifically and across tissue types within individual plants [4,43,44]. In vitro enzymatic assays demonstrated that PyCCR1, PyCCR2, PyCCR5, and PyCCR6 from *P. yunnanensis* catalyze the conversion of *p*-coumaroyl-CoA, sinapoyl-CoA, and feruloyl-CoA to their corresponding aldehydes (*p*-coumaraldehyde, sinapaldehyde, and coniferaldehyde, respectively). Our results clearly establish the catalytic activity of the identified PyCCRs, providing foundational knowledge of their roles in *P. yunnanensis*. While we have focused on the systematic identification and qualitative functional characterization of these genes, detailed steady-state kinetic modeling remains a target for subsequent biochemical investigations, which will further deepen the functional understanding of these conifer enzymes. Cinnamyl alcohol dehydrogenase (CAD) catalyzes the reduction of *p*-coumaraldehyde, sinapaldehyde, and coniferaldehyde to their corresponding alcohols, the immediate precursors for lignin polymerization [45,46,47].

*P. yunnanensis* predominantly contains G-type lignin. We thus speculate that *p*-coumaraldehyde, or its downstream alcohol (*p*-coumaryl alcohol), may be integrated into the G-lignin biosynthesis pathway via a two-step modification: hydroxylation at the 3-carbon position, followed by O-methylation, ultimately yielding the G-type lignin precursors coniferaldehyde and coniferyl alcohol, respectively. These findings underscore the need for further in vivo studies to verify the physiological role of CCR in lignin biosynthesis in *P. yunnanensis*. Collectively, this study clarifies the functional diversity of CCRs in *P. yunnanensis*, providing a basis for elucidating their regulatory mechanisms.

Nine recombinant PyCCR proteins from *P. yunnanensis* were successfully expressed. In vitro enzymatic assays demonstrated that PyCCR1/2/5/6 exhibited catalytic activity, specifically converting cinnamoyl-CoA to cinnamaldehyde, whereas the remaining PyCCRs showed no detectable activity. Molecular docking analysis of *P. yunnanensis* CCRs protein models with various substrates revealed that the R(X)5K motif plays a crucial role in substrate binding by PyCCRs. The R(X)5K motif residues, along with adjacent residues, form a binding pocket that positions the substrate molecule and facilitates hydrogen bond formation with specific residues, revealing PyCCRs regional binding selectivity. Structural analysis suggests that PyCCRL7~12 likely lacks the R(X)5K motif and H(X)2K motif, resulting in the absence of catalytic activity and substrate binding capability. The identification of these key residues provides a novel strategy for future site-directed mutagenesis studies. However, a systematic kinetic analysis to determine their substrate preference and catalytic efficiency is required in future studies. Furthermore, although the current in vitro assays did not demonstrate catalytic activity for the CCR-like (PyCCRL) members, exploring whether they possess alternative biological functions in vivo represents a compelling avenue for future research [17].

### 4.3. Expression Pattern of CCRs Genes in P. yunnanensis

Lignin biosynthesis displays strict tissue-specific regulation [48,49,50,51], primarily occurring in highly lignified tissues [27,35]. Numerous studies have demonstrated that modulating CCR expression directly alters both lignin content and monomer composition [52,53,54]. Extensive research has demonstrated tissue-specific CCR expression patterns, with predominant transcript accumulation in stems and roots across diverse plant species [55,56]. Tissue-specific expression profiling revealed differential expression patterns among *PyCCR1* to *PyCCRL12* in *P. yunnanensis*. The majority of *PyCCRs* exhibited predominant expression in buds and stems, suggesting their potential role in catalyzing substrate conversion to cinnamaldehyde derivatives in these lignification-active tissues, with subsequent transport to other tissues for lignin monomer biosynthesis. Within a single plant species, significant variations are observed in the composition and spatial distribution of lignin among different tissues. Moreover, even within a specific tissue region, the lignin composition undergoes dynamic changes during plant growth and development. This heterogeneity primarily arises from the spatiotemporal differential expression of genes involved in the lignin biosynthesis pathway [57]. Among the characterized PyCCRs, PyCCR1 and PyCCR6 exhibited high expression levels in stems and roots, whereas PyCCR2 and PyCCR5 were predominantly expressed in fruits. Based on these expression patterns, it is speculated that PyCCR1 and PyCCR6 primarily catalyze the formation of cinnamoyl-CoA in stems and roots, whereas PyCCR2 and PyCCR5 predominantly catalyze this reaction in fruits to generate cinnamaldehyde. CCR homologs typically exhibit peak expression during active plant growth phases, with particularly abundant transcripts detected in stem, root, and xylem tissues. Lignin deposition is essential for mechanical support and vascular development during plant growth [14,58]. RT-qPCR analysis demonstrated tissue-specific expression profiles of *PyCCRs*, indicative of their functional specialization during plant development. The functional characterization of some *PyCCRs* may be limited by the heterologous *E. coli* expression system (BL21/pET30a (+)), non-native induction conditions (IPTG concentration and temperature), and inherent substrate selectivity. Future investigations employing eukaryotic expression systems, physiologically relevant induction conditions, and expanded substrate screening would enable complete functional elucidation of *PyCCR* genes.

## 5. Conclusions

This study identified twelve CCR genes in *P. yunnanensis* and classified them into CCR (PyCCR1~6) and CCR-like (PyCCRL7~12) clades. Among the nine recombinant proteins successfully expressed, four (PyCCR1, PyCCR2, PyCCR5, and PyCCR6) were confirmed by in vitro assays to catalyze the conversion of *p*-coumaroyl-CoA, feruloyl-CoA, and sinapoyl-CoA. Combined molecular docking and structural analysis revealed that the conserved R(X)5K motif might be a key determinant of catalytic function, explaining the functional divergence between the two clades. The tissue-specific expression patterns of PyCCRs further support their specialized roles in lignification. Collectively, these findings provide both a mechanistic framework for understanding lignin biosynthesis and specific genetic targets for improving wood properties in *P. yunnanensis*.

## Figures and Tables

**Table 1:** The characteristic of the 12 *PyCCRs* in *P. yunnanensis*.

Gene ID	Gene Name	ORF	Relative Molecular Mass/kDa
g2800_i1	*PyCCR1*	975 bp	35.63
g36102_i0	*PyCCR2*	1026 bp	37.96
g26496_i0	*PyCCR3*	1026 bp	38.17
g15984_i8	*PyCCR4*	1050 bp	39.17
g35923_i1	*PyCCR5*	966 bp	35.93
g32859_i0	*PyCCR6*	993 bp	36.81
g28353_i1	*PyCCRL7*	963 bp	35.19
g17061_i0	*PyCCRL8*	795 bp	29.69
g13735_i2	*PyCCRL9*	1074 bp	39.82
g33456_i0	*PyCCRL10*	948 bp	35.20
g7090_i3	*PyCCRL11*	945 bp	34.49
g17854_i1	*PyCCRL12*	945 bp	34.54

## Data Availability

The gene and protein sequences used in this study to analyze the cinnamoyl-CoA reductase (CCR) gene family of *P. yunnanensis* are available in the NCBI database (GenBank accession numbers: PyCCR1 PX845401, PyCCR2 PX845402, PyCCR3 PX845403, PyCCR4 PX845404, PyCCR5 PX845405, PyCCR6 PX845406, PyCCR7 PX845407, PyCCR8 PX845408, PyCCR9 PX845409, PyCCR10 PX845410, PyCCR11 PX845411, PyCCR12 PX845412).

## Supplementary Materials

The following supporting information can be downloaded at: https://www.mdpi.com/article/10.3390/plants15152253/s1, Table S1. Reverse transcription reaction system. Table S2. Primer sequences for CCR gene amplification in *P. yunnanensis*. Table S3. RT-PCR reaction system. Table S4. RT-PCR reaction procedure. Table S5. Homologous recombination reaction system. Table S6. Reaction system of BSA standard protein solution. Table S7. High Performance Liquid Chromatography (HPLC) analytical procedure. Table S8. RT-qPCR reaction procedure. Table S9. RT-qPCR reaction system. Table S10. RT-qPCR primer information. Table S11. Summary of subcellular localization predictions for PyCCRs proteins. Figure S1. PCR amplification of *PyCCR* genes from *P. yunnanensis*. Lane M: DNA marker (with sizes indicated in bp). The expected fragment sizes are as follows: *PyCCR1* (975 bp), *PyCCR2* (1026 bp), *PyCCR3* (1026 bp), *PyCCR4* (1050 bp), *PyCCR5* (966 bp), *PyCCR6* (993 bp), *PyCCRL7* (963 bp), *PyCCRL8* (795 bp), *PyCCRL9* (1074 bp), *PyCCRL10* (948 bp), *PyCCRL11* (945 bp), *PyCCRL12* (945 bp). All amplified fragments matched the expected sizes, with no non-specific bands observed. Figure S2. Colony PCR verification of the recombinant prokaryotic expression vectors in *E. coli* DH5α. The agarose gel electrophoresis shows the target DNA fragments amplified directly from the transformed DH5α competent cells to confirm the successful cloning of *PyCCR* genes. Lane M: DNA marker; Lanes 1–12: PCR products from transformed bacterial liquids showing the expected sizes of *PyCCR* inserts. Figure S3. Expression of recombinant protein of PyCCR1~12-6×His M. Protein molecular weight marker (10–180 kDa); CK. pET30a (+)-BL21 empty vector control; 1. Uninduced control (28 °C, 10 h); 2–6. IPTG-induced (0.2–1.0 mM) at 28 °C for 10 h (OD600 = 0.6–0.8); 7. Soluble fraction after sonication; 8. Insoluble fraction after sonication. Figure S4. Expression of recombinant protein of PyCCR4-6×His, PyCCRL8-6×His, PyCCRL10-6×His and PyCCRL11-6×His under 16 °C. M is a protein Marker (10~180 kDa); CK. Empty carrier pET30a(+)-BL21; 1. Without IPTG induction, 16 °C for 10 h; 2. 0.2 mM IPTG, 16 °C for 10 h (OD600 = 0.6~0.8); 3. Supernatant after ultrasound cell fragmentation; 4~5. Precipitation after cell fragmentation by ultrasound. Figure S5. Expression of recombinant PyCCR1~12-6×His proteins. Lane M. Protein marker (10–180 kDa); CK. pET30a(+) empty vector control; 1. Uninduced control; 2. 0.2 mM IPTG induction; 3. 0.4 mM IPTG induction; 4. 0.6 mM IPTG induction; 5. 0.8 mM IPTG induction; 6. 1.0 mM IPTG induction; 7. Supernatant after ultrasonication; 8. Pellet after ultrasonication. Red arrow indicates the target protein band. Figure S6. The content of purified protein was determined by BSA method. Figure S7. Molecular docking of PyCCR2/4 with feruloyl-CoA. (A) and (E) Binding sites of PyCCR2 (off-white) and PyCCR4 (light green) for feruloyl-CoA. Feruloyl-CoA substrate is shown as pink stick models. Key residues are displayed as stick models with cyan markers. Hydrogen bonds are indicated by yellow dashed lines. (C) and (D) Surface representations of PyCCR4 and PyCCR2 with bound feruloyl-CoA. (B) and (F) Interactions between feruloyl-CoA (stick model) and catalytic active-site residues of PyCCR2 and PyCCR4 (spherical and stick models). (PyCCR2 and PyCCR3 conform to the homology model.). Figure S8. Molecular docking of PyCCR5/6 with feruloyl-CoA. (A) and (E) The binding sites of PyCCR5 and PyCCR6 for feruloyl-CoA are highlighted in yellow and pink, respectively. The feruloyl-CoA substrate (pink) is depicted as a stick model. Key residues are displayed as stick models, with cyan markers indicating their positions. Hydrogen bonds are represented as yellow dashed lines. (C) and (D) Surface representations of PyCCR5 and PyCCR6 in complex with feruloyl-CoA. (B) and (F) Molecular interactions between feruloyl-CoA (stick model) and the catalytic active site residues of PyCCR5 and PyCCR6 (spherical and stick models). Figure S9. Molecular docking of PyCCRL7 with feruloyl-CoA. (A) Binding sites of PyCCRL7 (light pink) for feruloyl-CoA. Feruloyl-CoA substrate is shown as pink stick models. Key residues are displayed as stick models with cyan markers. Hydrogen bonds are indicated by yellow dashed lines. (C) Surface representations of PyCCRL7 with bound feruloyl-CoA. (B) Interactions between feruloyl-CoA (stick model) and catalytic active-site residues of PyCCRL7 (spherical and stick models).
