# Supplementary material for: Identification and In Vitro Functional Characterization of the CCR Gene Family Reveals Their Regulatory Roles in Lignin Biosynthesis of Pinus yunnanensis
Source: Plants (Basel). 2026 Jul 23;15(15):2253. doi: 10.3390/plants15152253 (PMC13468098; doi:10.3390/plants15152253)
Supplement: Supplementary file 1 [file plants-15-02253-s001.zip › plants-4379471-supplementary.pdf]

Supplementary Material

Identification and In Vitro Functional Characterization of the *CCR* Gene Family Reveals Their Regulatory Roles in Lignin Biosynthesis of *Pinus yunnanensis*

Jun Liu [1,2], Heze Wang [1,2], Jianhong Chang [1,2], Aiqin Yao [1,2] and Junrong Tang [1,2,*]

1 Key Laboratory for Forest Resources Conservation and Use in the Southwest Mountains of China, Ministry of Education, Southwest Forestry University, Kunming 650224, China; liujun0102123@163.com (J.L.); hezewang@swfu.edu.cn (H.W.); m15752798665@163.com (J.C.); 15184968530@163.com (A.Y.)

2 Yunnan Provincial Key Laboratory for Conservation and Utilization of In-Forest Resource, Southwest
Forestry University, Kunming 650224, China

* Correspondence: tjrzy2016@swfu.edu.cn

Supplementary Table

Table S1. Reverse transcript reaction system.

Reagents	Reaction system
Total RNA	500ng
PyCCRs-F（10 μM）	2μL
PyCCRs-R（10 μM）	2μL
cDNA	1μL
ddH2O	Up to 20μL
Total	20μL

Table S2. Primer sequences for CCR gene amplification in *P. yunnanensis.*

Gene Name	Primer sequence（5‘→3’）	Note
PyCCR1	F: GCCATGGCTGATATCggatccATGACTGCAGGTAAACAAACC<br>R: TGCGGCCGCAAGCTTgtcgacTCACTTGGAGATATGCCCTTTTTC	
PyCCR2	F: GCCATGGCTGATATCggatccATGAAGCAAACGGAAAGAAC<br>R: TGCGGCCGCAAGCTTgtcgacTCAGATCCTAGAAAACAGCGAG	
PyCCR3	F: GCCATGGCTGATATCggatccATGAAAGAAACGAACAGAACAGT<br>R: TGCGGCCGCAAGCTTgtcgacTCAGATCCTAGACAATAGCGG	
PyCCR4	F: GCCATGGCTGATATCggatccATGATATCAGATCAAACGACGC<br>R: TGCGGCCGCAAGCTTgtcgacTCACATCCTCGCCAATATTGA	RT-PCR
PyCCR5	F: GCCATGGCTGATATCggatccATGAGATCAGATCAAACTGTCTG<br>R: TGCGGCCGCAAGCTTgtcgacTCAGAGATAGTGAAGAAAGCCC	
PyCCR6	F: GCCATGGCTGATATCggatccATGGGACCAGATCAAATGC<br>R: TGCGGCCGCAAGCTTgtcgacCTAGTGGAGAAAGCCCTTG	
PyCCRL7	F: GCCATGGCTGATATCggatccATGGATGGCTATCTTGAAGAAAAT<br>R: TGCGGCCGCAAGCTTgtcgacCTATGCCAGCTCTCCAGAA	
PyCCRL8	F: GCCATGGCTGATATCggatccATGTTGAATTCTTTTACAGAGGAAACA	

	R: TGCGGCCGCAAGCTTgtcgacTTATGCCAGCTCCCCAGA	
PyCCRL9	F: GCCATGGCTGATATCggatccATGGCGATCGTGAAGAC<br>R: TGCGGCCGCAAGCTTgtcgacTCACTCACTCTCACGTCTG	
PyCCRL10	F: GCCATGGCTGATATCggatccATGGCTGTTTGTCGACAAA<br>R: TGCGGCCGCAAGCTTgtcgacTTAAACTTGGTTCCCTGATTGC	
PyCCRL11	F: GCCATGGCTGATATCggatccATGCCGGTGTTTCCTTC<br>R: TGCGGCCGCAAGCTTgtcgacTTACAGTTCGCTGCTTACGG	
PyCCRL12	F: GCCATGGCTGATATCggatccATGCTGGTGTTTCCTTCTG<br>R: TGCGGCCGCAAGCTTgtcgacTCACAGTTCGCTGCTTAGG	
pET30a (+)	F: TAATACGACTCACTATAGGGGAATT<br>R: CAAAAAACCCCTCAAGACCC	Colony PCR

Table S3. RT-PCR Reaction system.

Component Name	Volume (μL)
2×Phanta Max Master Mix (Dye Plus)	25
PyCCRs-F（10 μM）	2
PyCCRs-R（10 μM）	2
cDNA	1
ddH2O	Up to 50
Total	50

Table S4. RT-PCR Reaction procedure.

Steps	Reaction temperature	Reaction time	PCR cycles
Initial denaturation step	95 °C	5 min	
Denaturation	95 °C	15 s	
Annealing	56 °C	15 s	38
Extension	72 °C	90 s	
Complete Extension	72 °C	10 min	

Table S5. Homologous recombination reaction system.

Component Name	Volume (μL)
5×CE II Buffer	4
Exnase II	2
Linearization carrier	X
Purpose segment	Y
ddH_2O	Up to 20
Total	20

Table S6. Reaction System of BSA standard protein solution.

Component Name	0	1	2	3	4	5	6	7
1 mg/mL BSA（μL）	0	10	20	30	40	50	60	70
ddH2O（μL）	100	90	80	70	60	50	40	30
Bradford reagent（mL）	3	3	3	3	3	3	3	3
BSA concentration（mg/mL）	0	0.1	0.2	0.3	0.4	0.5	0.6	0.7

Table S7. High Performance Liquid Chromatography（HPLC）analytical procedure.

Time（min）	HPLC-grade acetonitrile (mobile phase A)	0.1% (v/v) phosphoric acid in water (mobile phase B)	Flow rate（mL/min）	Column temperature（°C）
0	5%	95%		
10	5%	95%		
25	15%	85%	1.0	30
45	25%	75%		
50	95%	5%		

Table S8. RT-qPCR reaction procedure.

Steps	Reaction temperature	Reaction time	PCR cycles
Initial denaturation step	95 °C	30 s	
PCR cycling reaction	95 °C	10 s	40
	58 °C	30 s	
Dissociation curve	95 °C	15 s	
	60 °C	1 min	
	95 °C	15 s	

Table S9. RT-qPCR reaction system.

Component Name	Volume (μL)
cDNA templates from different tissues	1
Primer F	0.5
Primer R	0.5
2×Taq Pro Universal SYBR qPCR Master Mix	10
ddH2O	Up to 20
Total	20

Table S10. RT-qPCR primer information.

Primer name	Primer sequence（5`→3`）
Actin-4-F	GGGAGACCAGGCAATGAAGAAGATC
Actin-4-R	CAATGGCATACCGAATGGCAACAAC
QPyCCR1-F	GCCGCTATTTATGTGCCGAGAG
QPyCCR1-R	GCTTCCCATCTTCCTTGACCTTG
QPyCCR2-F	CAAGAGCTTAGTCGCAGTTATCCG
QPyCCR2-R	GCCTCCATCACATTCTTCGTTCC
QPyCCR3-F	CGTGGCTTGTTAAGATGCTCCTC
QPyCCR3-R	CTCCTTCCAACGCTCTAAGATGTG
QPyCCR-4-F	AGGTGGAAGTATGAGCATCTGAGAG
QPyCCR-4-R	TGAGAACTGAAGCCATGTGAAAGAC
QPyCCR5-F	CTGTGCGGCGAATGTAGTCTC
QPyCCR5-R	AACTTGTATGGCTGCTTCCTTGG
QPyCCR6-F	ACTGTTGGAGCGATTTGGATTACTG
QPyCCR6-R	TCGGATTCACCACTGCCATCTC
QPyCCRL7-F	TGGTGTTCACTTCCTCCGTATCG
QPyCCRL7-R	ATTCAAGATTGGTCCAGCACTTCTC
QPyCCRL8-F	GAGTAGATTACGAGGTTCAGAGTGC
QPyCCRL8-R	ATGGCGGACAGAGAGGAAGTG
QPyCCRL9-F	AGGGTGGGAGAGATGGAGGAG
QPyCCRL9-R	TCACAAAGAGCAGCAGTAGAATCAG

QPyCCRL10-F	GCAGCAGATGGATTGGACTATCG
QPyCCRL10-R	TCGCTCATCATAGGCAGATTCATTC
QPyCCRL11-F	GGTACGCATTAGCCAAGACACTG
QPyCCRL11-R	ACCAGGTCCCACAATCAGAGATG
QPyCCRL12-F	GGTACGCATTAGCCAAGACACTG
QPyCCRL12-R	TGCCAGGTCCTACAATCAGAGATG

Table S11. Summary of subcellular localization predictions for PyCCRs proteins.

Protein name	Subcellular localization forecast
PyCCR1	39.1% Cytosol, 17.4% Mitochondria, 17.4% Nucleus, 13.0% Endoplasmic Reticulum, 4.3% Peroxisomes, 4.3% Golgi Apparatus, 4.3% Secretory System Vesicles
PyCCR2	34.8% Cytosol, 34.8% Mitochondria, 21.7% Nucleus, 4.3% Cytoskeleton, 4.3% Secretory System Vesicles
PyCCR3	39.1% Mitochondria, 30.4% Cytosol, 21.7% Nucleus, 4.3% Secretory System Vesicles, 4.3% Cytoskeleton
PyCCR4	69.6% Cytosol, 13.0% Mitochondria, 13.0% Nucleus, 4.3% Cytoskeleton
PyCCR5	52.2% Cytosol, 13.0% Cytoskeleton, 13.0% Mitochondria, 13.0% Nucleus, 8.7% Peroxisomes
PyCCR6	65.2% Cytosol, 26.1% Nucleus, 8.7% Mitochondria
PyCCRL7	47.8% Cytosol, 21.7% Nucleus, 8.7% Mitochondria, 4.3% Cytoskeleton, 4.3% Secretory System Vesicles, 4.3% Vacuole, 4.3% Endoplasmic Reticulum, 4.3% Golgi Apparatus
PyCCRL8	69.6% Cytosol, 13.0% Nucleus, 8.7% Mitochondria, 4.3% Secretory System Vesicles, 4.3% Endoplasmic Reticulum
PyCCRL9	69.6% Cytosol, 13.0% Nucleus, 8.7% Mitochondria, 4.3% Vacuole, 4.3% Secretory System Vesicles
PyCCRL10	56.5% Cytosol, 17.4% Nucleus, 17.4% Mitochondria, 4.3% Cytoskeleton, 4.3% Secretory System Vesicles
PyCCRL11	87% Cytosol, 8.7% Mitochondria, 4.3% Secretory System Vesicles
PyCCRL12	87% Cytosol, 8.7% Mitochondria, 4.3% Cell wall

Supplementary Figure

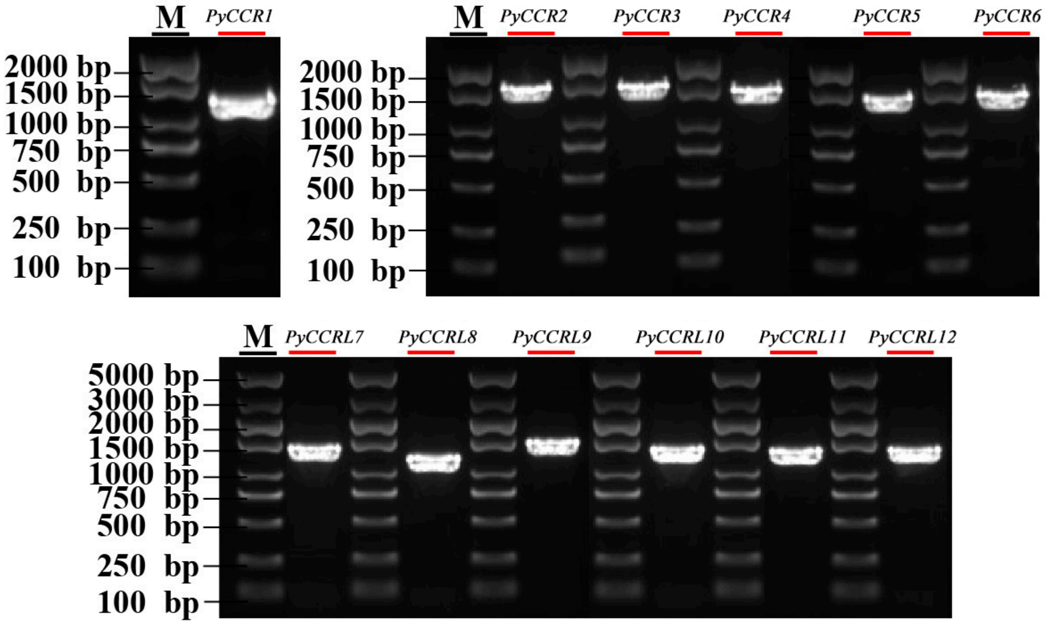

Figure S1. PCR amplification of *PyCCR* genes from *P. yunnanensis*. Lane M: DNA marker (with sizes indicated in bp). The expected fragment sizes are as follows: *PyCCR1* (975 bp), *PyCCR2* (1026 bp), *PyCCR3* (1026 bp), *PyCCR4* (1050 bp), *PyCCR5* (966 bp), *PyCCR6* (993 bp), *PyCCRL7* (963 bp), *PyCCRL8* (795 bp), *PyCCRL9* (1074 bp), *PyCCRL10* (948 bp), *PyCCRL11* (945 bp), *PyCCRL12* (945 bp). All amplified fragments matched the expected sizes, with no non-specific bands observed.

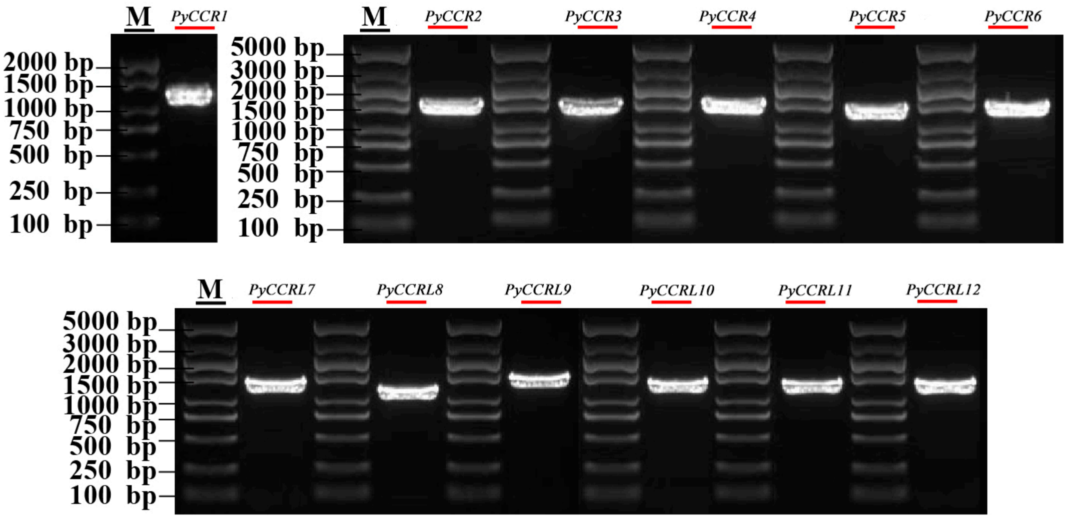

Figure S2. Colony PCR verification of the recombinant prokaryotic expression vectors in *E. coli* DH5α. The agarose gel electrophoresis shows the target DNA fragments amplified directly from the transformed DH5α competent cells to confirm the successful cloning of *PyCCR* genes. Lane M: DNA marker; Lanes 1–12: PCR products from transformed bacterial liquids showing the expected sizes of *PyCCR* inserts.

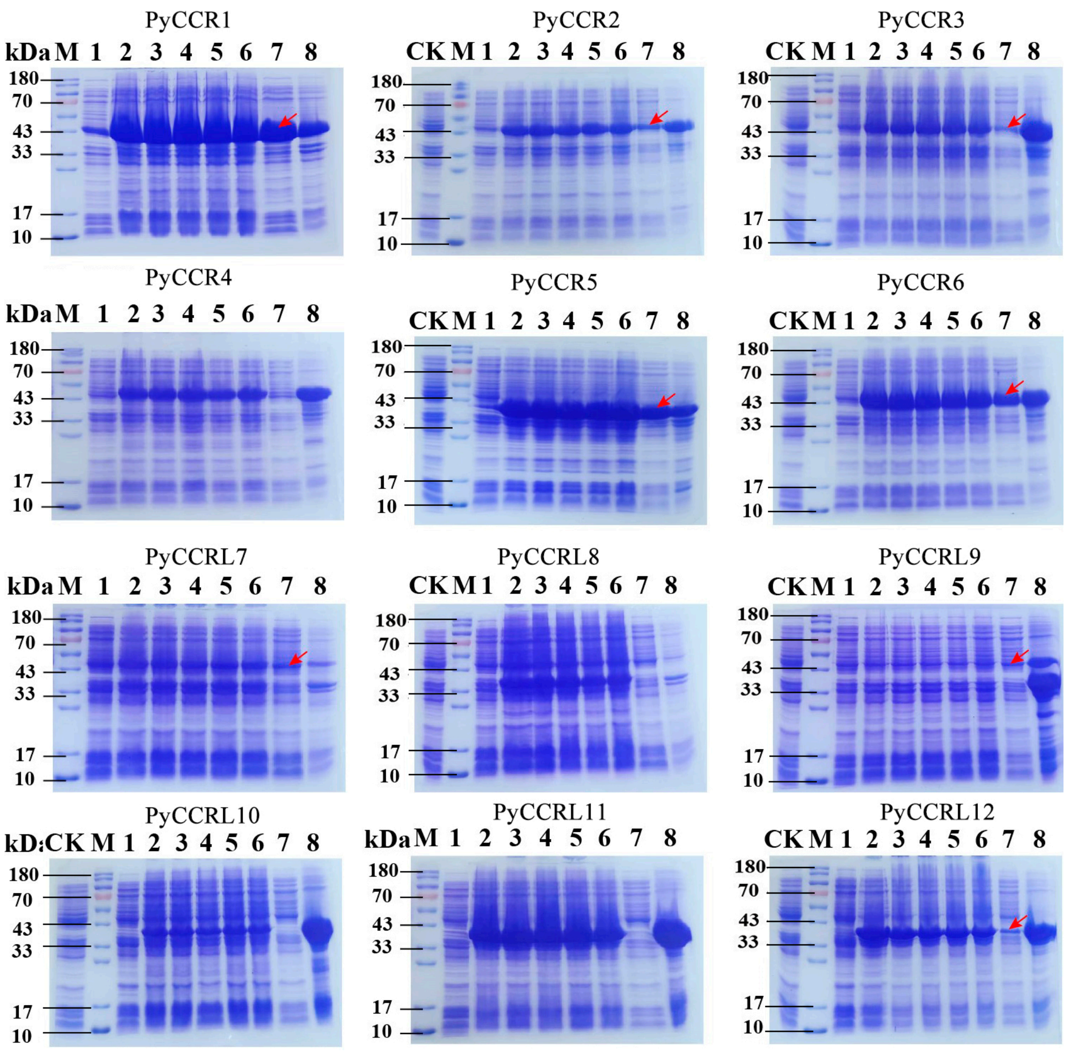

Figure S3. Expression of recombinant protein of PyCCR1~12-6×His M. Protein molecular weight marker (10-180 kDa); CK. pET30a (+)-BL21 empty vector control; 1. Uninduced control (28°C, 10 h); 2-6. IPTG-induced (0.2-1.0 mM)at 28°C for 10 h (OD600 = 0.6-0.8); 7. Soluble fraction after sonication; 8. Insoluble fraction after sonication.

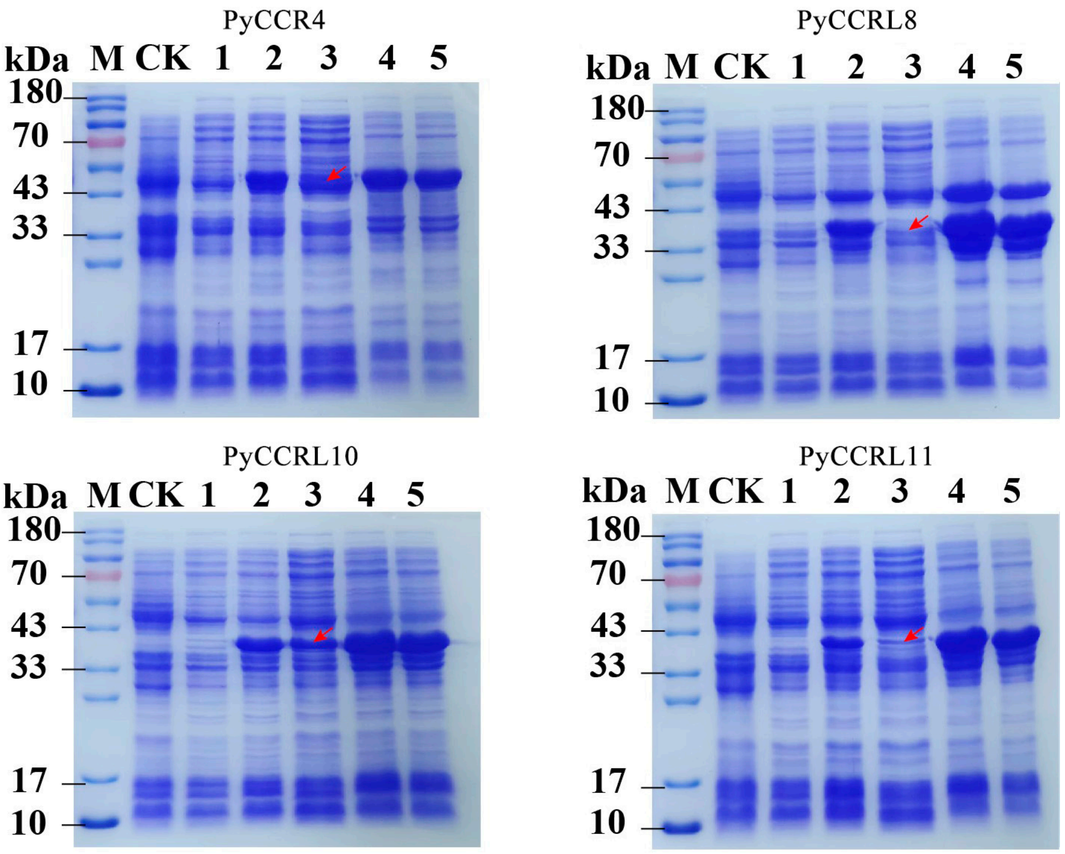

Figure S4. Expression of recombinant protein of PyCCR4-6×His, PyCCRL8-6×His, PyCCRL10-6×His and PyCCRL11-6×His under 16 °C. M is a protein Marker（10~180 kDa）; CK. Empty carrier pET30a(+)-BL21; 1. Without IPTG induction, 16 °C for 10 h; 2. 0.2 mM IPTG, 16 °C for 10 h（OD600 = 0.6~0.8）; 3. Supernatant after ultrasound cell fragmentation; 4~5. Precipitation after cell fragmentation by ultrasound.

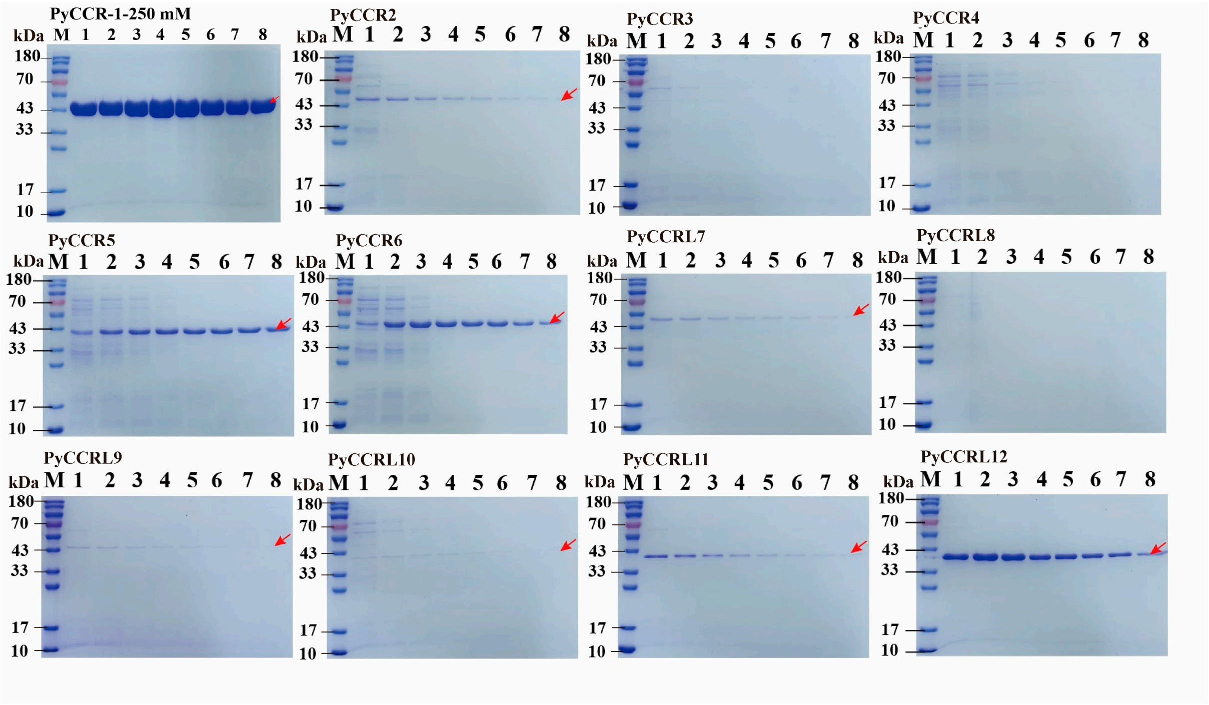

Figure S5. Expression of recombinant PyCCR1~12-6×His proteins. Lane M. Protein marker (10-180 kDa); CK. pET30a(+) empty vector control; 1. Uninduced control; 2. 0.2 mM IPTG induction; 3. 0.4 mM IPTG induction; 4. 0.6 mM IPTG induction; 5. 0.8 mM IPTG induction; 6. 1.0 mM IPTG induction; 7. Supernatant after ultrasonication; 8. Pellet after ultrasonication. Red arrow indicates the target protein band.

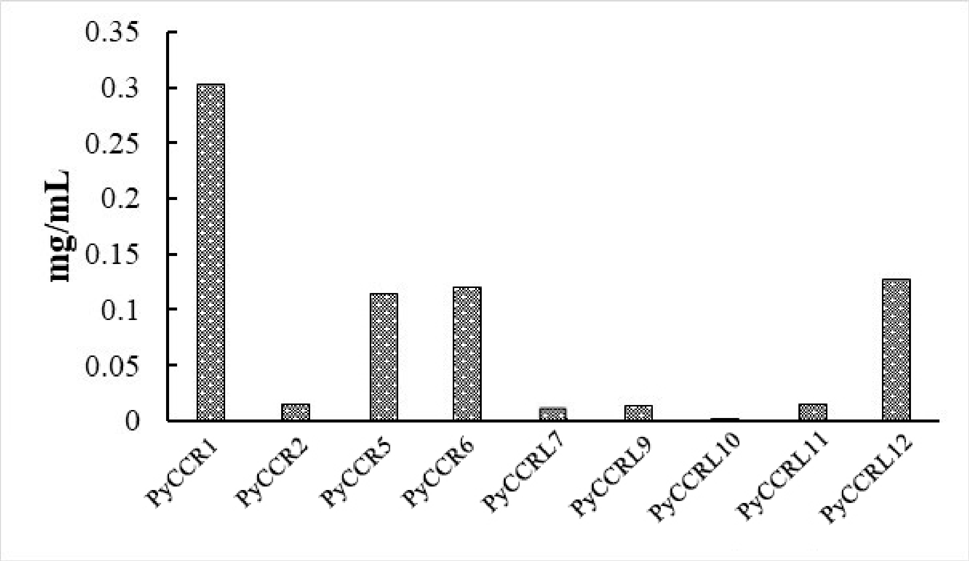

Figure S6. The content of purified protein was determined by BSA method.

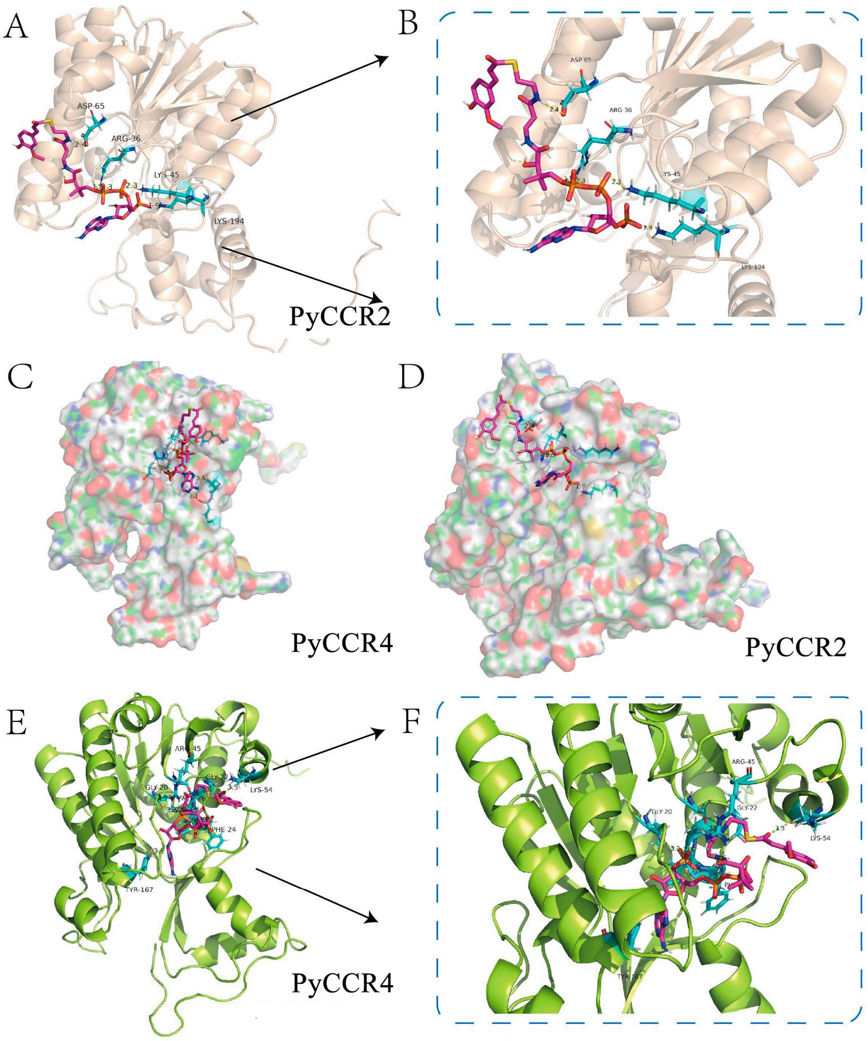

Figure S7. Molecular docking of PyCCR2/4 with feruloyl-CoA.
(A) and (E) Binding sites of PyCCR2 (off-white) and PyCCR4 (light green) for feruloyl-CoA. Feruloyl-CoA substrate is shown as pink stick models. Key residues are displayed as stick models with cyan markers. Hydrogen bonds are indicated by yellow dashed lines. (C) and (D) Surface representations of PyCCR4 and PyCCR2 with bound feruloyl-CoA. (B) and (F) Interactions between feruloyl-CoA (stick model) and catalytic active-site residues of PyCCR2 and PyCCR4 (spherical and stick models). (PyCCR2 and PyCCR3 conform to the homology model.)

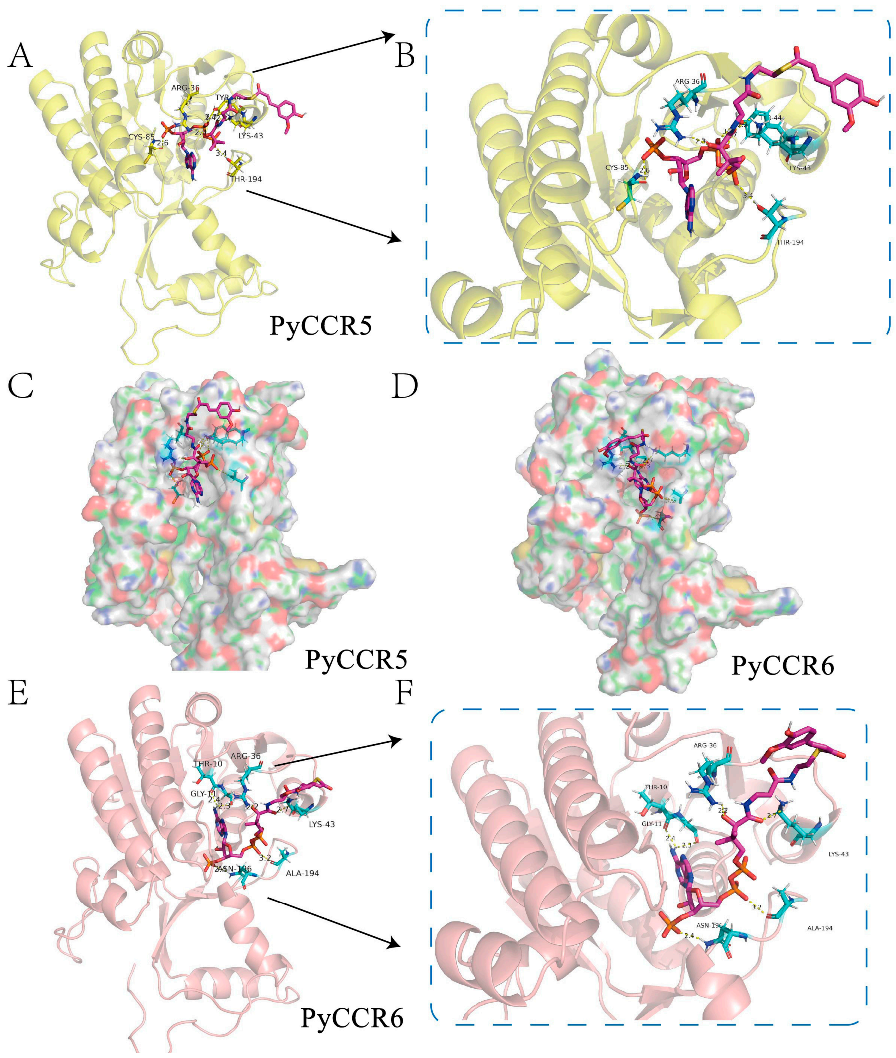

Figure S8. Molecular docking of PyCCR5/6 with feruloyl-CoA.
(A) and (E) The binding sites of *PyCCR5* and *PyCCR6* for feruloyl-CoA are highlighted in yellow and pink, respectively. The feruloyl-CoA substrate (pink) is depicted as a stick model. Key residues are displayed as stick models, with cyan markers indicating their positions. Hydrogen bonds are represented as yellow dashed lines. (C) and (D) Surface representations of *PyCCR5* and *PyCCR6* in complex with feruloyl-CoA. (B) and (F) Molecular interactions between feruloyl-CoA (stick model) and the catalytic active site residues of *PyCCR5* and *PyCCR6* (spherical and stick models).

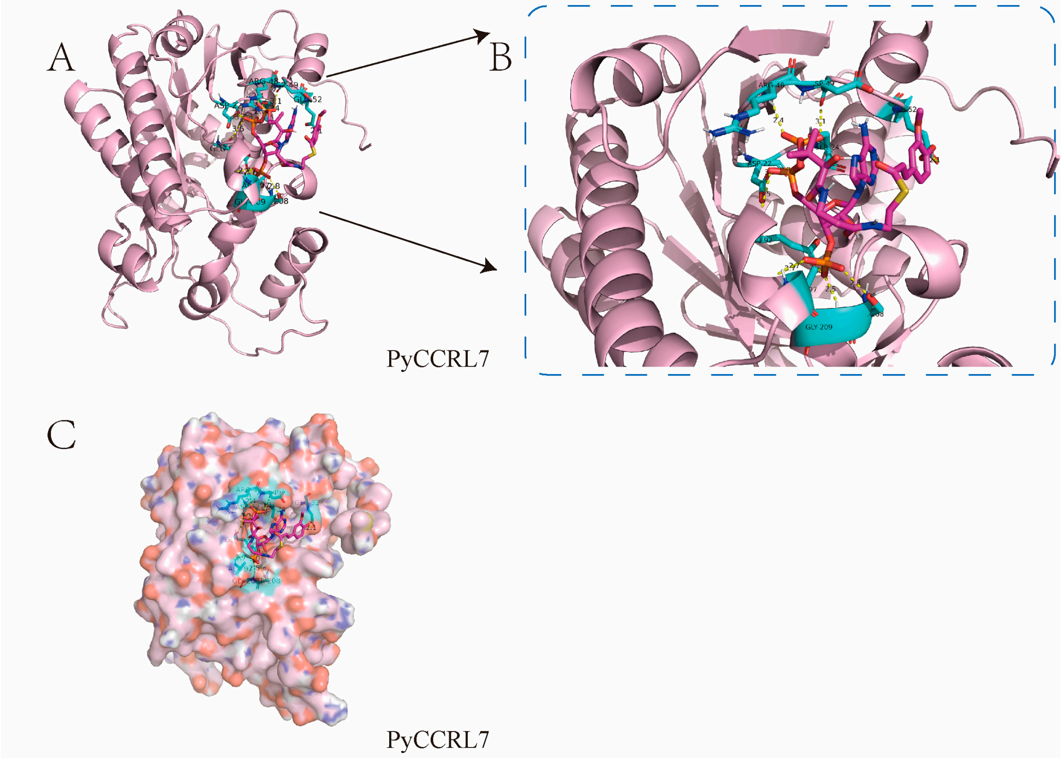

Figure S9. Molecular docking of PyCCRL7 with feruloyl-CoA.
(A) Binding sites of PyCCRL7 (light pink) for feruloyl-CoA. Feruloyl-CoA substrate is shown as pink stick models. Key residues are displayed as stick models with cyan markers. Hydrogen bonds are indicated by yellow dashed lines. (C) Surface representations of PyCCRL7 with bound feruloyl-CoA. (B) Interactions between feruloyl-CoA (stick model) and catalytic active-site residues of PyCCRL7 (spherical and stick models).
